# Protein expression profiles indicative for drug resistance of non-small cell lung cancer

**DOI:** 10.1038/sj.bjc.6600463

**Published:** 2002-08-01

**Authors:** M Volm, R Koomägi, J Mattern, T Efferth

**Affiliations:** German Cancer Research Centre, Heidelberg, Germany; Virtual Campus Rhineland-Palatinate, P.O. Box 4380, 55033 Mainz, Germany

**Keywords:** non-small cell lung carcinomas, resistance factors, hierarchical cluster analysis

## Abstract

Data obtained from multiple sources indicate that no single mechanism can explain the resistance to chemotherapy exhibited by non-small cell lung carcinomas. The multi-factorial nature of drug resistance implies that the analysis of comprising expression profiles may predict drug resistance with higher accuracy than single gene or protein expression studies. Forty cellular parameters (drug resistance proteins, proliferative, apoptotic, and angiogenic factors, products of proto-oncogenes, and suppressor genes) were evaluated mainly by immunohistochemistry in specimens of primary non-small cell lung carcinoma of 94 patients and compared with the response of the tumours to doxorubicin *in vitro*. The protein expression profile of non-small cell lung carcinoma was determined by hierarchical cluster analysis and clustered image mapping. The cluster analysis revealed three different resistance profiles. The frequency of each profile was different (77, 14 and 9%, respectively). In the most frequent drug resistance profile, the resistance proteins P-glycoprotein/MDR1 (MDR1, ABCB1), thymidylate-synthetase, glutathione-S-transferase-π, metallothionein, O^6^-methylguanine-DNA-methyltransferase and major vault protein/lung resistance-related protein were up-regulated. Microvessel density, the angiogenic factor vascular endothelial growth factor and its receptor FLT1, and ECGF1 as well were down-regulated. In addition, the proliferative factors proliferating cell nuclear antigen and cyclin A were reduced compared to the sensitive non-small cell lung carcinoma. In this resistance profile, FOS was up-regulated and NM23 down-regulated. In the second profile, only three resistance proteins were increased (glutathione-S-transferase-π, O^6^-methylguanine-DNA-methyltransferase, major vault protein/lung resistance-related protein). The angiogenic factors were reduced. In the third profile, only five of the resistance factors were increased (MDR1, thymidylate-synthetase, glutathione-S-transferase-π, O^6^-methylguanine-DNA-methyltransferase, major vault protein/lung resistance-related protein).

*British Journal of Cancer* (2002) **87**, 251–257. doi:10.1038/sj.bjc.6600463 www.bjcancer.com

© 2002 Cancer Research UK

## 

Lung cancer remains a major cause of morbidity and mortality in Western countries. The majority of bronchogenic carcinomas can be classified into four histological types: small cell lung carcinomas, squamous cell lung carcinomas, adenocarcinomas, and large cell lung carcinomas. Histological features, ultra-structure, clinical course, and response to therapy indicate that small cell lung cancer is a separate entity. The other three histological subtypes are referred to as non-small cell lung cancer (NSCLC). While small cell lung carcinomas are among the most drug sensitive tumours, NSCLC are frequently resistant to drug therapy and obtaining a complete response is rare. Therefore, the drug resistance in patients receiving chemotherapy alone or when combined with radiotherapy represents a major problem in cancer treatment of patients with NSCLC.

Data obtained from multiple sources indicate that no single mechanism can explain the resistance to therapy exhibited by NSCLC. The resistance mechanisms are numerous and diverse (Valeriote and van Putten, 1975; Bradley *et al*, 1988; Kelley *et al*, 1988; Scanlon and Kashani-Sabet, 1988; Whelan *et al*, 1989; Huot *et al*, 1991; Kaina *et al*, 1991, Volm *et al*, 1991; Koomägi *et al*, 1995; Hickman, 1996; Volm and Mattern, 1996; Volm and Rittgen, 2000). They depend on the detoxifying capacity of the cells, repair capacity, drug delivery, cell proliferation and many other factors. Therefore, a key challenge is to determine the relative quantitative contributions of each of these mechanisms to the drug resistant phenotype. In order to understand the complex network of genes in clinically relevant drug resistance, it is rather not sufficient to investigate single genes. Holistic analyses of an entire battery of genes conferring drug resistance may be more appropriate to gain insight into the full potential of unresponsive tumours.

In an endeavour to cope with the complex resistance mechanisms, in this investigation 40 cellular parameters were evaluated in specimens of NSCLC of 94 patients. The purpose of this study was to determine the resistance profiles of NSCLC by hierarchical cluster analysis.

## MATERIALS AND METHODS

### Patients and tumours

Ninety-four patients (83 men, 11 women) with previously untreated NSCLC were admitted to this study. All patients were surgically treated at the Chest Hospital in Heidelberg-Rohrbach. The morphological classification of the carcinomas was conducted according to the WHO specifications. Of the carcinomas, 48 were squamous carcinomas, 34 were adenocarcinomas and 12 were large cell carcinomas. All patients were staged at the time of their surgery according to the guidelines of the American Joint Committee on Cancer. Sixteen patients had stage I, 12 patients stage II and 66 patients had stage III tumours. The mean age of the patients was 59 years.

### Detection of tumour resistance *in vitro*

Most of the patients were treated by surgical procedures alone. Only a small group of patients were treated by combined surgical and radiation treatment or chemotherapy but the additional radiation treatment and chemotherapy had no significant effect on patient survival time (*P*>0.1) and on the cluster analysis. For determining the resistance of tumours we used a short-term *in vitro* test that has been described previously (Volm *et al*, 1979, 1988a). Its basic feature is measurement of changes in the incorporation of radioactive nucleic acid precursors into tumour cells after addition of doxorubicin. We found that anthracyclines (e.g. doxorubicin) can be used as reference compound for multiple resistance. Tumours were defined as being sensitive or resistant depending on whether nucleotide uptake was inhibited by more or less than 65% at a concentration of doxorubicin of 10 μg ml^−1^. This threshold was based on prior clinical correlation (Volm *et al*, 1979).

### Immunohistochemistry

The previously described method was used to detect the proteins in formalin-fixed and paraffin-embedded specimens (Volm *et al*, 1991, 1998; Koomägi and Volm, 1999). Briefly, formalin-fixed and paraffin-embedded tissue were deparaffinised. After pre-incubation with hydrogen peroxide and protein blocking solution, the primary antibodies were applied for 16 h at 4°C. After incubation with secondary antibodies, the streptavidin biotinylated peroxidase complex was added and the peroxidase activity visualised with 3-amino-9-ethylcarbazole. Counter-staining was performed with haematoxylin. Both negative and positive controls were conducted. Negative controls were prepared by omitting the primary antibodies and by substituting irrelevant antibodies for the primary antibodies. The specificity of the reactions were proved by Western blots. Three observers independently evaluated the results from the immunohistochemical staining without having any prior knowledge of an individual patient's clinical data. The evaluations agreed in 90–95% of the samples. The other specimens (5–10%) were re-evaluated and then classified according to the classification most frequently given by the observers. To evaluate the protein expression, the staining intensity or the staining intensity and the percentages of positive cells were determined.

A part of the antibodies for the detection of resistance-proteins were gifts from several laboratories, others were commercially available. The appropriate concentrations were found by additional experiments. Anti-glutathione S-transferase-π (GSTP1) was kindly donated by Dr K Satoh (University School of Medicine, Hirosaki, Japan) anti-DNA topoisomerase II (TOP2A) by Dr L Liu (John Hopkins Oncology Center, Baltimore, MD, USA); anti-metallothionein (MT) by Dr PC Huang (Johns Hopkins University, Baltimore, MD, USA); anti-thymidylate synthetase (TYMS) by Dr B Yates (Burroughs Welcome, Research Triangle Park, Cornwallis USA); and anti-O^6^-methylguanine-DNA-methyl-transferase (MGMT) by Dr B Li (University Singapore, Singapore). The anti-P-glycoprotein (MDR1, ABCB1) antibody JSB-1 was obtained from Sanbio (Uden, Netherlands); anti-catalase (CAT) from Calbiochem (La Jolla, CA, USA); anti-heat shock protein 70 (HSPA1A) from Dako Diagnostika (Hamburg, Germany), and anti-major vault protein/lung resistance-related protein (MVP/LRP) from Dunn Labortechnik (Asbach, Germany). For detection of the proliferative activity, anti-cyclin A (CCNA), anti-CDK2 and anti-CDK4 were obtained from Santa Cruz Biotechnology (Heidelberg, Germany). Anti-cyclin D1 (CCND) was from Calbiochem/Novabiochem (Baden-Soden, Germany) and PCNA from Dianova (Hamburg, Germany). The antibody for detection of the apoptotic factor FAS/CD95 (TNFRSF6) was from Immunotech (Hamburg, Germany) and anti-FAS ligand (TNFSF6) and anti-caspase 3 (CASP3) from Santa Cruz Biotechnology. The antibodies for staining of angiogenic factors were anti-VEGF obtained from Dianova, anti-tissue factor (TF, F3) from Biodesign (Kennebunk, MA, USA), anti-bFGF, anti-FGFR and anti-FLT1 from Santa Cruz Biotechnology. Anti-angiostatin was purchased from Oncogene Research Products (Cambridge, MA, USA). Anti-PD-ECGF (ECGF1) was a gift from Dr Tanaka (Nippon Roche Research Centre, Kamakura, Japan). For the detection of the proto-oncogene and suppressor gene products we used the following antibodies: c-K-*ras* (KRAS), c-H-*ras* (HRAS), c-N-*ras* (NRAS), FOS, JUN, MYC, ERBB1, ERBB2, RB1 and p53 (TP53). All antibodies for the above mentioned proto-oncogene and suppressor gene proteins were from Dianova. Anti-BCL2 was from Oncogene Research Products and anti- p16^INK4A^ (CDKN2A) was from Santa Cruz Biotechnology.

Anti-NM23 was obtained from Novocastra (Newcastle-upon-Tyne, UK) and anti-HIF-1α (HIF1A) and anti-HIF-1β (HIF1B) from Novus Biologicals (Littleton, CO, USA).

### Measurement of micro-vessel density

Blood vessels were highlighted by staining endothelial cells for factor VIII using the strepatavidin-biotin peroxidase complex method. Micro-vessel density was determined by counting labelled capillaries in the areas of highest vascularisation within the tumour mass. Individual counts of blood vessels were determined by light microscopy in a 250× field as described earlier (Weidner *et al*, 1991).

### Assessment of apoptosis

Apoptotic cell death was detected with a non-radioactive 3′-end DNA labelling technique which used the *in situ* cell death detection kit (TUNEL reaction). The procedure was described earlier (Stammler and Volm, 1996).

### DNA cell cycle analysis by flow cytometry

A mixture of propidium iodide and 4′-6-diamidino-2-phenylindole was applied simultaneously with RNAse after methanol fixation and protease digestion of single cell suspensions (Volm *et al*, 1988b). Flow cytometry analysis was undertaken with an ICP-22 (Phywe, Göttingen, Germany). Peripheral blood leukocytes from healthy donors were used as a calibration standard for DNA diploidy. Parallel measurements, both including and omitting the standard, were performed. The cell cycle analysis was performed using integrated Gaussian fittings. A computerised subtraction of exponentially decreasing corrections beginning with the peak of cellular debris was included in the evaluation program. The cell cycle analysis was omitted in cases that exhibited interspersed cell populations.

### Statistical analysis

The doxorubicin resistance parameter (short-term test) is expressed as a percentage of the controls. These values define a ‘sensitive’ group (less than 65% at a concentration of 10^−2^ mg ml^−1^ doxorubicin) and a ‘resistant’ group (not less than 65%). This classification is used later on to examine whether other parameters show significant relationships.

The immunohistochemical parameters were evaluated on either a binary scale (‘no reaction’ or ‘reaction’, coded as ‘−’ or ‘+’) or an ordinal scale ‘no reaction’, ‘weak’, ‘moderate’ or ‘strong reaction’ (coded as ‘−’, ‘+’, ‘++’ or ‘+++’). The apoptotic indices, micro-vessel density and the cell cycle phases were counting variables and, therefore, the median values were used. To find out the predictive value of these parameters with respect to doxorubicin resistance Fisher's exact tests were performed. The results of these tests with *P*-values <0.1 are summarised in Table 1

**Table 1:**
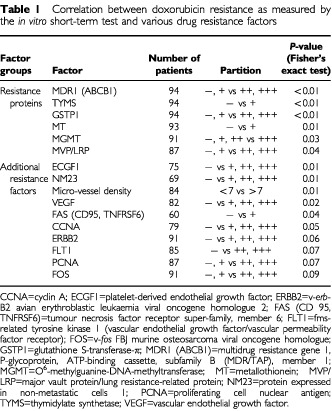
Correlation between doxorubicin resistance as measured by the *in vitro* short-term test and various drug resistance factors

. The parameters with a *P* value greater than 0.1 were not analysed any further.

Hierarchical cluster analysis is an explorative statistical method and aims to group at first sight heterogeneous objects into clusters of homogeneous objects. Objects are classified by calculation of distances according to the closeness of between-individual distances. All objects are assembled into a cluster tree (dendrogram). Thus, objects with tightly related features appear together, while the separation in the cluster tree increases with progressive dissimilarity. Cluster analyses applying average- or complete-linkage methods were done by means of the WinSTAT program (Kalmia Company). Missing values are automatically omitted by the program and the closeness of two joined objects was calculated by the number of data points they contained. In order to calculate distances of all variables included in the analysis, the program automatically standardises the variables by transforming the data with a mean=0 and a variance=1. To construct clustered-image maps (CIM), two dendrograms were related to each other. The 16 resistance factors were cluster-ordered on the basis of their expression pattern across the 94 NSCLC. Thus, resistance parameters with most nearly identical pattern appear side by side on the x-ordinate. *Vice versa*, the 94 NSCLC were cluster-ordered across the resistance factors. Tumours with most identical expression patterns of resistance factors appear side by side on the y-co-ordinate.

## RESULTS

**Figure 1:**
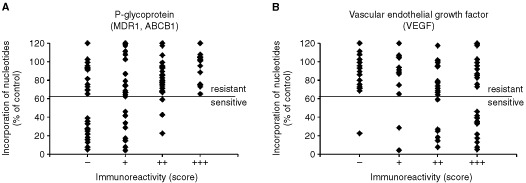
Relationship between the drug response (doxorubicin) as determined by the *in vitro* short term test (ordinate) and the immunohistochemical reaction of P-glycoprotein/MDR1 and VEGF. The intensity of immunostaining (negative, weak, moderate, high) is specified as −, +, ++, +++

The objective of this investigation was to evaluate the profile of protein expression involved in resistant NSCLC. For this reason, the expressions of 40 factors in primary NSCLC of 94 patients were determined and correlated with the data obtained by the *in vitro* resistance test. Examples are given in Figure 1

. Of the 94 patients with NSCLC 70 patients had resistant carcinomas, whereas 24 patients revealed sensitive carcinomas. As visible in Figure 1, P-glycoprotein is positively correlated with resistance while VEGF is negatively correlated.

In a first step, we analysed the relationships of the expression levels of the 40 factors with sensitivity/resistance by Fisher's exact tests. Of the investigated resistance proteins P-glycoprotein/MDR1 (MDR1, ABCB1), glutathione-S-transferase-π (GSTP1), metallothionein (MT), O^6^-methyl-guanine-DNA-methyltransferase (MGMT), lung resistance-related protein (MVP/LRP), thymidylate synthetase (TYMS), DNA-topoisomerase II (TOP II), catalase (CAT) and heat shock protein 70 (HSP 70) six proteins (MDR1, TYMS, GSTP1, MT, MGMT and MVP/LRP) showed a relationship to sensitivity/resistance (*P*<0.1) (Table 1). These six proteins were increased in resistant NSCLC. Of the proliferative factors analysed (PCNA, cyclin A, cyclin D, the cyclin-dependent kinases cdk2 and cdk4, cell cycle phases) only PCNA and cyclin A (CCNA) exhibited a relationship to sensitivity/resistance and were down-regulated in resistant carcinomas. Of the angiogenic proteins tested, vascular endothelial growth factor (VEGF), its receptor FLT1 and platelet-derived endothelial growth factor (ECGF1) showed a relationship to sensitivity/resistance. These factors were reduced in resistant carcinomas. This was confirmed by the reduction of micro-vessel density in resistant tumours (Table 1). Such relationship was not found with basic fibroblast growth factor (bFGF), its receptor FGFR and with angiostatin. No relationship exists between the expression of tissue factor and the resistance of tumours. Of the pro-apoptotic factors investigated FAS/CD95, FAS ligand, and caspase-3 only FAS/CD95 was significantly reduced in resistant NSCLC. The anti-apoptotic factor BCL-2 showed no obvious relationship to sensitivity/resistance of NSCLC. There is huge evidence that proto-oncogenes and suppressor genes are implicated in resistance of tumours. Therefore, we investigated the products of c-*fos*, c-*jun*, c-*myc*, c-*erb*B1, c-*erb*B2, c-K-*ras*, c-H-*ras*, c-N-*ras*, rb, p53, nm23 and p16^INK4a^. Of these proteins only FOS, ERBB2 and NM23 revealed a correlation to sensitivity/resistance of NSCLC. Finally, hypoxia causes a wide range of responses in tumours. Therefore, we assessed whether hypoxia-inducible factors (HIF) may also play a role in the resistance of NSCLC. Both HIF-1α and HIF-1β did not reveal a relationship to sensitivity/resistance of NSCLC. In Table 1, the 16 factors are listed which showed relationships to the doxorubicin response of NSCLC (*P*<0.1) The parameters with *P*-values greater than 0.1 were not analysed any further.

**Figure 2:**
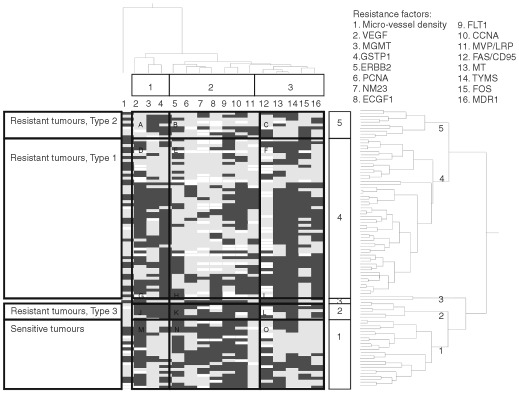
Dendrograms and clustered image map obtained by hierarchical cluster analysis (complete linkage method). The dendrogram on the right shows the clustering of 94 NSCLC and the dendrogram on the top of 16 resistance factors. Light fields indicate ‘not expressed’ and dark fields indicate ‘expressed’ according to the partition for each resistance factor shown in Table 2. Missing values are depicted in white

This kind of analysis, however, does not allow an insight to complex expression profiles which constitute resistant or sensitive tumour phenotypes. Therefore, we decided to perform hierarchical cluster analyses which may be more suited to unravel the full potential of such data sets for an integrated understanding of drug resistance. We subjected these 16 proteins to hierarchical cluster analysis, in order to find out expression profiles indicative for drug resistance of NSCLC (Figure 2

, dendrogram on the right).

We divided the dendrogram into five clusters and correlated them with the *in vitro* resistance data which were not included as parameter into the cluster analysis (Table 2

**Table 2:**
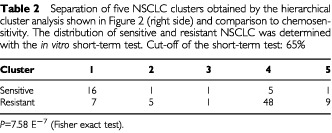
Separation of five NSCLC clusters obtained by the hierarchical cluster analysis shown in Figure 2 (right side) and comparison to chemosensitivity. The distribution of sensitive and resistant NSCLC was determined with the *in vitro* short-term test. Cut-off of the short-term test: 65%

). Sensitive and resistant NSCLC were separated in the clusters (*P*=7.58 E^−7^). Cluster 1 (*n*=23 cases) is enriched with sensitive carcinomas (78%), while cluster 2 (*n*=6 cases), cluster 4 (*n*=53 cases) and cluster 5 (*n*=10 cases) were enriched with resistant carcinomas (83, 89 and 90% respectively). These clusters showed no relationships to other clinical parameters (stage, survival).

The mean values of the 16 parameters of all investigated carcinomas of the clusters were measured and the ratios of resistant/sensitive clusters determined (Table 3

**Table 3:**
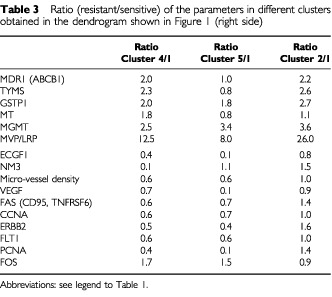
Ratio (resistant/sensitive) of the parameters in different clusters obtained in the dendrogram shown in Figure 1 (right side)

). The cluster analysis revealed that three different resistance profiles exist. The frequency of the resistance profiles are different (cluster 4: 77%, cluster 5: 14%, cluster 2: 9%).

In the most frequent drug resistance profile (cluster 4) all resistance proteins investigated (MDR1, TYMS, GSTP1, MT, MGMT, MVP/LRP) were up-regulated. Micro-vessel density and the angiogenic factors ECGF1, VEGF, FLT1 were down-regulated. Additionally, the proliferative factors PCNA and CCNA were reduced. The apoptotic factor FAS/CD95 was less expressed than in sensitive carcinomas. Of the proto-oncogenes and suppressor genes FOS was up-regulated, while NM23 and ERBB2 were down-regulated.

The analysis of the carcinomas of cluster 5 shows that only three of the six investigated resistance proteins were up-regulated (GSTP1, MGMT, MVP/LRP). Again, micro-vessel density was reduced and the angiogenic factors (VEGF, ECGF1) were more down-regulated than those of the carcinomas in cluster 4. The proliferative and apoptotic factors were reduced. In contrast to the carcinomas in cluster 4, NM23 was not changed.

The evidence of the third resistance profile (cluster 2) is limited because of the small number of carcinomas in this cluster. Only five resistance proteins (MDR1, TYMS, GSTP1, MGMT, MVP/LRP) were increased in comparison to sensitive tumours, while the other resistance factors revealed only marginal changes.

Next, we performed a cluster analysis using the 16 resistance factors and found a dendrogram which can be separated into three areas (Figure 2, top). The first area obtained three parameters (VEGF, MGMT and GSTP1). The second area contained in their majority proliferation- or angiogenesis-regulating proteins (e.g. ERBB2, PCNA, ECGF1, FLT1, and CCNA). In the third area were genes known to contribute to drug resistance and apoptosis (FAS/CD95, MT, TYMS, FOS, MDR1).

As shown in Figure 2, the CIM can be subdivided into several areas (A to O). The sensitive NSCLC (CIM areas M and O) were characterised by a less frequent expression of drug resistance proteins than resistant tumours of CIM areas D and F. On the other hand, these resistant tumours revealed a lower expression of proliferation/angiogenesis-regulating proteins (CIM area E) compared to sensitive tumours (CIM area N). The tumours grouped in CIM areas A to C were resistant as well, but they showed a different protein expression profile compared to the resistant tumours of CIM areas D to F. These NSCLC exhibited a rather low expression of drug resistance genes (except MGMT and GSTP1; CIM area A) and proliferation/angiogenesis-regulating proteins (CIM areas B and C). Resistant tumours of CIM areas J to L consisted of only few cases, but they seem to be distinct from the other resistant tumours of CIM areas A to I with a strong expression of all proteins investigated. Cluster 3 consisted of only two tumours and are thus less informative (CIM areas G to I). The clustering was independent of stage of tumours (*P*>0.9) and of survival of patients (*P*>0.7).

## DISCUSSION

The advances in molecular and cellular biology have opened new avenues for the characterisation of drug resistant tumours. Protein and RNA expression are in the centre of interest. Protein detection by immunohistochemistry and RNA detection by Northern blotting or reverse transcription-polymerase chain reaction (RT–PCR) may be more suited for clinical application than assays such as Western blotting or RNAse protection assay. In this study, we used mainly immunohistochemical assays to analyse expression profiles of proteins of NSCLC.

To analyse the effect of doxorubicin, we used the nucleotide incorporation test (*in vitro* short-term test), because the patients were operated only. Previously, different *in vitro* test systems (tissue culture assay, colony assay and short term test) to monitor the resistance were compared and similar results with all three methods were obtained (Volm *et al*, 1987). In a clinical study, 55 of 57 tumours that were resistant in the short-term test were also clinically progressive (Group for Sensitivity Testing of Tumors (KSST), 1981). Several authors have confirmed this consistency between *in vitro* test results and the clinical results (Auner *et al*, 1989; Khoo *et al*, 1989). These evidences let us believe that the *in vitro* short term is suitable to determine the resistance of NSCLC.

There is striking evidence that a wide variety of drug resistance mechanisms are operational in clinically relevant drug resistance. In this analysis, several resistance proteins and additional resistance-related factors were changed in resistant NSCLC in comparison to sensitive carcinomas. The reason for this concomitant expression of different factors may be the result of inducing a cascade of resistance-related gene products that can be triggered by environmental factors such as smoking (Volm *et al*, 1991, 1999; Mattern *et al*, 1998). Another possible explanation is that lung cancer is mostly detected at a relatively late stage. These carcinomas are mainly hypoxic and the vascular network that supplies oxygen and nutrients is substantially lower. In fact, in our study the micro-vessel density and several angiogenic factors were reduced in resistant NSCLC.

Data obtained in a variety of systems show that the proliferation may also be an important resistance factor (Kohn *et al*, 1994; Cordon-Cardo, 1995). Indeed, we found in the present study that the proliferative factors were decreased in resistant NSCLC. Apoptosis or programmed cell death is an important homeostatic mechanism that balances cell production with cell death (Thompson, 1995). In our analysis, FAS/CD95 was also lower in resistant NSCLC compared to sensitive carcinomas.

Because indications exist that proto-oncogenes and tumour suppressor genes may be implicated in tumour resistance (Burt *et al*, 1988), we examined the expressions of the proteins of some of these factors. In our analysis, the expressions of ERBB2, NM23, and FOS played an important role in resistant NSCLC. ErbB2 encodes a protein that acts as a transmembrane growth factor receptor and showed a similar reaction as the proliferative factors CCNA and PCNA on the response. NM23 which is a putative metastasis suppressor gene has also been shown to be associated with cell proliferation and apoptosis (Venturelli *et al*, 1995). FOS and JUN constitute the AP-1 protein complex which binds specifically to the AP-1 binding site in promotors of resistance genes (Teeter *et al*, 1991). Though this is known for a decade, the full relevance of AP1 and FOS may have been underestimated as of yet.

The multi-facetted nature of drug resistance implies that the analysis of entire expression profiles may predict drug resistance with higher accuracy and may be superior to single gene expression studies. By means of clustered image mapping (CIM) we found four different protein expression profiles. One expression profile was characterised by low expression of drug resistance proteins and high expression of proliferation/angiogenesis-regulating proteins (CIM areas M to O). This expression profile was indicative for sensitive tumours. Three different types of resistant tumours were visible by means of CIM. Type 1 revealed a high expression of drug resistance genes and a lower expression of proliferation/angiogenesis-regulating proteins (CIM areas D to F). The majority of tumours belonged to this group indicting that this expression profile is the most important for NSCLC. Type 2 is characterised by a low or intermediate expression of drug resistance genes and a rather low expression of proliferation/angiogenesis-regulating proteins. Thus, this type of resistance seemed predominately to be due to a decreased proliferative and angiogenetic capacity of tumours. Type 3 consisted of few resistant tumours with high expression of drug resistance as well as proliferation/angiogenesis-associated proteins. As a high expression of proliferative and angiogenetic markers contribute rather to chemosensitivity, the high expression of drug resistance proteins may be superior for the expression of a resistant phenotype. This analysis gives a clue that the molecular architecture of drug resistance is far more complex as estimated as of yet and that different types of expression profiles are responsible for the development of drug resistance. The high prediction of *in vitro* resistance (83–90%) which resulted from the combined cluster calculation of 16 proteins is clearly superior to the prediction of resistance by any single of these proteins. It can, thus, be inferred from the CIM data that chemotherapy would be more beneficial to patients with sensitive tumours of CIM areas M to O than to patients with resistant tumours of CIM areas A to F and J to L).

These results may have important implications for diagnosis and therapy of NSCLC. Our data envision the definition of characteristic resistance gene profile able to predict clinical response to chemotherapy. Although the statistical probability of the resistance profile of NSCLC may be known, the clinical response of the individual patient remains still uncertain. However, the recently thriving micro-array technology may allow simultaneous analyses of thousands of genes of a single patient in a single experiment (Shim *et al*, 1998; Golup *et al*, 1999; Sgroi *et al*, 1999; Ono *et al*, 2000; Ross *et al*, 2000; Scherf *et al*, 2000). The idea to generate a predictive test for lung cancer patients is gaining ground with such novel approaches. This concept embodies to develop individual cytostatic treatment schedules for each patient. On the other hand, it has been suggested that a minimal set of about 10 to 50 genes may provide more robust results than sets of thousands of genes (Wooster, 2000). It is still open to discussion, whether results obtained from large-scale gene expression experiments may be of limited utility for routine diagnostics and whether the wealth of genes not involved in resistance produce a high background which masks the view of the relevant genes. The resistance proteins and the resistance-related factors which we found in the present analysis may be suitable parameters to predict treatment response of NSCLC. We believe that these parameters together with hierarchical cluster analysis as appropriate tool to identify responding or non-responding tumours are determinants which will ease the development of a predictive test methodology for NSCLC.

## References

[bib1] AunerHPetruEHofmannHMAPickelHPürstnerP1989 In vitro chemosensitivity testing in the treatment of ovarian cancer Arch Gynecol Obstet 246 227231 2619337 10.1007/BF00934523

[bib2] BradleyGJurankaPFLingV1988 Mechanisms of multidrug resistance Biochim Biophys Acta 948 87128 2899442 10.1016/0304-419x(88)90006-6

[bib3] BurtRKGarfieldSJohnsonKThorgeirssonS1988 Transformation of rat liver epithelial cells with v-H-ras or v-H-raf causes expression of MDR1, glutathione S-transferase-π and increased resistance to cytotoxic chemicals Carcinogenesis 9 23292332 2903802 10.1093/carcin/9.12.2329

[bib4] Cordon-CardoC1995 Mutation of cell cycle regulators. Biological and clinical implications for human neoplasia (review) Am J Pathol 147 545560 7677168 PMC1870966

[bib5] GolupTRSlonimDKTamayoPHuardCGaasenbeekMMesirovJPCollerHLohMLDowningJRCaligiuriMABloomfieldCDLanderES1999 Molecular classification of cancer: class discovery and class prediction by gene expression monitoring Science (Washington) 286 531537 10521349 10.1126/science.286.5439.531

[bib6] Group for Sensitivity Testing of Tumors (KSST)1981 In vitro short-term test to determine the resistance of human tumors to chemotherapy Cancer 48 21272135 7197581 10.1002/1097-0142(19811115)48:10<2127::aid-cncr2820481002>3.0.co;2-2

[bib7] HickmanJA1996 Apoptosis and chemotherapy resistance Eur J Cancer 6 921926 10.1016/0959-8049(96)00080-9 8763333

[bib8] HuotJRoyGLambertHChretienPLandryJ1991 Increased survival after treatments with anticancer agents of Chinese hamster cells expressing the human Mr 27.000 heat shock protein Cancer Res 51 245252 1913647

[bib9] KainaBFritzGMitraSCoquerelleT1991 Transfection and expression of human O^6^-methylguanine-DNA-methyltransferase (O^6^-MGMT) cDNA in Chinese hamster cells: The role of MTMT in protection against the genotoxic effects of alkylating agents Carcinogenesis 12 18571867 1657427 10.1093/carcin/12.10.1857

[bib10] KelleySLBasuATeicherBAHackerMPHamerDHLazoJ1988 Overexpression of metallothionein confers resistance to anticancer drugs Science (Washington) 241 18131815 3175622 10.1126/science.3175622

[bib11] KhooSKHurstTWeebMJDickieGKearslyJParsonsPGMackayE1989 Clinical value of in vitro drug sensitivity testing based on short-term effects on DNA and RNA metabolism in ovarian cancer J Surg Oncol 41 201205 2747246 10.1002/jso.2930410314

[bib12] KohnKWJackmanJO'ConnorP1994 Cell cycle control and cancer chemotherapy Cell Biochem 54 440452 10.1002/jcb.240540411 8014193

[bib13] KoomägiRMatternJVolmM1995 Up-regulation of resistance-related proteins in human lung tumors with poor vascularization Carcinogenesis 16 21292133 7554065 10.1093/carcin/16.9.2129

[bib14] KoomägiRVolmM1999 Expression of Fas (CD95/Apo-1) and Fas-ligand in lung cancer, its prognostic and predictive relevance Int J Cancer (Pred Oncol) 84 239243 10.1002/(sici)1097-0215(19990621)84:3<239::aid-ijc7>3.0.co;2-s 10371340

[bib15] MatternJKoomägiRVolmM1998 Smoking-related increase of O^6^-methylguanine-DNA-methyltransferase expression in human lung carcinomas Carcinogenesis 19 12471250 9683184 10.1093/carcin/19.7.1247

[bib16] OnoKTanakaTTsunodaTKitaharaOOkamotoAOchiaiKTakagiTNakamuraY2000 Identification of cDNA microarray of genes involved in ovarian carcinogenesis Cancer Res 60 50075011 11016619

[bib17] RossDTScherfUEisenMBPerouCMReesCSpellmanPIyerVJeffreySSvan de RijnMWalthamMPergamenschikowALeeJCLashkariDShalonDMyersTGWeinsteinJNBotsteinDBrownPO2000 Sytematic variation in gene expression patterns in human cancer cell lines Nat Genet 24 227235 10700174 10.1038/73432

[bib18] ScanlonKJKashani-Sabet1988 Elevated expression of thymidylate synthase cycle genes in cisplatin-resistant human ovarian carcinoma A 2780 cells Proc Natl Acad Sci USA 85 650653 3422447 10.1073/pnas.85.3.650 PMC279612

[bib19] ScherfURossDTWalthamMSmithLHLeeJKTanabeLKohnKWReinholdWCMyersTGAndrewsDTScudieroDAEisenMBSausvilleEAPommierYBotsteinDBrownPOWeinsteinJN2000 A gene expression database for the molecular pharmacology of cancer Nat Genet 24 236244 10700175 10.1038/73439

[bib20] SgroiDCTengSRobinsonGLeVangieRHudsonJRElkahlounAG1999 In vivo gene expression profile analysis of human breast cancer progression Cancer Res 59 56565661 10582678

[bib21] ShimCZhangWRheeCHLeeJH1998 Profiling of differentially expressed genes in human primary cervical cancer by complementary DNA expression array Clin Cancer Res 4 30453050 9865919

[bib22] StammlerGVolmM1996 Apoptosis in non-small cell lung cancer as related to drug resistance and prognosis Apoptosis 1 9599

[bib23] TeeterLDEckersbergTTsaiYKuoMT1991 Analysis of the Chinese hamster p-glycoprotein/multidrug resistance gene pgp1 reveals that the AP-1 site is essential for full promoter activity Cell Growth Differ 2 429437 1661134

[bib24] ThompsonCB1995 Apoptosis in the pathogenesis and treatment of disease Science (Washington) 267 14561462 7878464 10.1126/science.7878464

[bib25] ValerioteFvan PuttenL1975 Proliferation-dependent cytotoxicity of anticancer agentsA reviewCancer Res 39 26192630 1098765

[bib26] VenturelliDMartinezRMelottiP1995 Overexpression of DR-nm23 gene, a protein encoded by a member of the nm23 gene family, inhibits granulocyte differentiation and induces apoptosis in 32Dc13 myeloid cells Proc Natl Acad Sci USA 92 74357439 7638209 10.1073/pnas.92.16.7435 PMC41354

[bib27] VolmMMatternJ1996 Resistance mechanisms and their regulation in lung cancer Crit Rev Oncogen 7 227244 10.1615/critrevoncog.v7.i3-4.50 9258604

[bib28] VolmMRittgenW2000 Cellular predictive factors for the drug response of lung cancer Anticancer Res 20 34493458 11131647

[bib29] VolmMKoomägiRMatternJ1999 Angiogenesis and cigarette smoking in squamous cell lung carcinomas: an immunohistochemical study of 28 cases Anticancer Res 19 333336 10226563

[bib30] VolmMKoomägiRRittgenW1998 Clinical implications of cyclins, cyclin-dependent kinases, RB and E2F1 in squamous cell lung carcinomas Int J Cancer (Pre Oncol) 79 294299 10.1002/(sici)1097-0215(19980619)79:3<294::aid-ijc15>3.0.co;2-8 9645354

[bib31] VolmMMatternJSamselB1991 Overexpression of P-glycoprotein and glutathione S-transferase- in resistant non-small cell lung carcinomas of smokers Br J Cancer 64 700704 1680367 10.1038/bjc.1991.384 PMC1977695

[bib32] VolmMDringsPHahnEWMatternJ1988a Prediction of the clinical chemotherapeutic response of the stage III lung adenocarcinoma patients by an in vitro short term test Br J Cancer 57 198200 3358911 10.1038/bjc.1988.42 PMC2246447

[bib33] VolmMEfferthTGüntherALathanB1987 Detection of murine S180 cells expressing a multidrug resistance phenotype using different in vitro test systems and a monoclonal antibody Arzneimittelforsch/Drug Res 37 862867 3314882

[bib34] VolmMWayssKKaufmannMMatternJ1979 Pretherapeutic detection of tumour resistance and the results of tumour chemotherapy Eur J Cancer 15 983993 488156 10.1016/0014-2964(79)90282-2

[bib35] VolmMHahnEWMatternJMüllerTMoykopfIWeberE1988b Five-year follow-up study of independent clinical and flow cytometric prognostic factors for the survival of patients with non-small cell lung carcinomas Cancer Res 48 29232928 2834052

[bib36] WeidnerNSempleJPWelchWRFolkmanJ1991 Tumor angiogenesis and metastasis- correlation in invasive breast carcinoma N Engl J Med 324 18 10.1056/NEJM199101033240101 1701519

[bib37] WhelanRDHHoskingLKTownsendAJCowanKHHillBT1989 Differential increases in glutathione S-transferase activities in a range of multidrug-resistant human tumor cell lines Cancer Commun 1 359365 2702041 10.3727/095535489820875057

[bib38] WoosterR2000 Cancer classification with DNA microarrays. Is less more? Trends Genet 16 327329 10904257 10.1016/s0168-9525(00)02064-3

